# Evaluation of Antidepressant Effects of Zhuyeqing Liquor Using *C. elegans* as a Model

**DOI:** 10.1002/fsn3.71349

**Published:** 2025-12-15

**Authors:** Xiaodong Cui, Jiahui Shen, Jing Yan, Chen Li, Jiao Li, Fang Li

**Affiliations:** ^1^ Institute of Biotechnology, Key Laboratory of Chemical Biology and Molecular Engineering of Ministry of Education Shanxi University Taiyuan China; ^2^ Shanxi Key Laboratory of Biotechnology Shanxi University Taiyuan China; ^3^ Xinghuacun College Shanxi University Taiyuan China; ^4^ School of Life Science Shanxi University Taiyuan China; ^5^ Shanxi Bethune Hospital, Shanxi Academy of Medical Sciences, Third Hospital of Shanxi Medical University, Tongji Shanxi Hospital Taiyuan China

**Keywords:** antidepressant effects, biomarker, *Caenorhabditis elegans*, egg‐laying, transcriptomic analysis, zhuyeqing liquor

## Abstract

Depression is a common psychiatric disorder in contemporary society, which severely impairs an individual's psychosocial functioning and quality of life. Zhuyeqing liquor (ZYQL), a well‐known traditional functional liquor in China, has not yet been evaluated for its antidepressant effects as a whole. In this research, we found that ZYQL stimulated egg‐laying in nematodes and improved their lifespan, pharyngeal pumping rate, and body bends. Subsequently, we conducted a transcriptomic analysis to identify biomarkers and underlying molecular mechanisms in fluoxetine‐treated groups. Ten biomarkers related to depression were screened and validated. These ten genes showed consistent responses to ZYQL and six traditional Chinese medicine ingredient extracts (TCMIE). Furthermore, these six TCMIEs reduced fat content and enhanced antioxidant activity. This study provides key biomarkers for evaluating the antidepressant effects of active ingredients and evaluates the antidepressant potential of ZYQL.

## Graphical abstract

*C. elegans* model was used to investigate the antidepressant effects and mechanism of ZYQL. Ten biomarkers related to depression were screened and validated in fluoxetine‐treated groups. This research is useful for formulating antidepressant beverages from ZYQL.

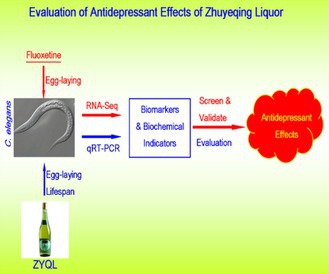

## 1 Introduction

Depression is a common psychiatric disorder that severely impairs an individual's psychosocial functioning and quality of life, with a high suicide rate, and remains a significant global concern (Diaz‐Camal et al. 2022). According to the latest report by the World Health Organization, approximately 350 million people worldwide suffer from depression (Trivedi 2020). Despite significant advances in understanding the pathophysiology of depression and the development of various medical treatments, safe and effective antidepressants are still lacking. Currently, common antidepressants can be categorized into six types: tricyclic antidepressants, monoamine oxidase inhibitors, selective 5‐HT reuptake inhibitors (SSRIs), norepinephrine reuptake inhibitors, tetracyclic antidepressants, and herbal medicines (Urits et al. 2019). The serotonin reuptake transporter (SERT) is the synaptic protein responsible for terminating a serotoninergic signal by removing or reabsorbing the released serotonin (5‐HT) (El‐Mallakh and Ali 2019). SSRIs can inhibit the removal of 5‐HT from the synaptic cleft, allowing more 5‐HT to interact with its specific receptors for a longer duration and with greater intensity (Silva et al. 2012). In clinical practice, SSRIs, such as fluoxetine, paroxetine, and sertraline, are often used to treat depression owing to their relatively few adverse effects.

In recent years, traditional Chinese medicine (TCM) has shown good efficacy in treating depression with minimal adverse effects. Various active ingredients from TCM play a key and indispensable role in the prevention and treatment of stress‐induced depression, including flavonoids, alkaloids, saponins, essential oils, terpenoids, and polyphenols (Dai et al. 2022). Numerous studies have suggested that Chinese medicine formulas (TCMFs) have the potential to significantly relieve depressive symptoms in depression and other disorders characterized by depressive‐like behaviors (C. Chen et al. 2023; Feng et al. 2020; Y.‐T. Wang et al. 2023). For example, St. John's Wort has been used as an antidepressant and is available as an over‐the‐counter herbal medicine in the United States (Brattström 2009; Ernst 1995). Moreover, Chinese herbal formulas such as Kai‐Xin‐San and Chai‐Hu‐Shu‐Gan‐San have been proven effective in treating and improving depression‐like symptoms (Cao et al. 2018; Hu et al. 2014; Y. Sun et al. 2018). Zhuyeqing liquor (ZYQL) is a famous traditional health liquor in China, composed of twelve classic Chinese medicinal herbs. These drugs are well‐known herbal medicines used for treating various diseases and as tonic medicines for thousands of years. ZYQL has shown beneficial effects in model organisms, such as antioxidant (Cui et al. 2023; H.‐y. Gao et al. 2014), anti‐fatigue (Han 2007), anti‐aging (Cui et al. 2023), hepatoprotective (Gao, Huang, et al. 2013), and immunoenhancing properties (Gao, Li, et al. 2013). Its components, such as *Gardenia jasminoides* Ellis (GJE), have good antidepressant effects (Hou et al. 2024), but the overall antidepressant potential of ZYQL has not yet been evaluated.

*Caenorhabditis elegans* ( *C. elegans* ), with its tiny size and simple anatomy, has become a model for aging, developmental biology, and toxicology research. Its basic neural system can integrate stimuli or signals from external environmental objects into behavioral output (Rani et al. 2023; Ruszkiewicz et al. 2018). The egg‐laying behavior of *C. elegans* has served as an important phenotype for the genetic analysis of neuronal signal transduction mechanisms (Shyn et al. 2003), including the behavioral effects of exogenous 5‐HT (Petrascheck et al. 2007). *C. elegans* provides faster and lower‐cost high‐throughput screening. Thus, it is favored as an ideal model organism for drug screening due to its relatively short lifecycle, ease of handling, ability to study whole animal responses, and complete sensory and neuromuscular systems.

In this paper, we used the *C. elegans* model to assess the antidepressant effects of ZYQL. Then, the transcriptomic analysis was performed to identify biomarkers and underlying molecular mechanisms in the SSRIs treatment groups. This study provides key biomarkers for evaluating the antidepressant effects of active ingredients and evaluates the overall antidepressant potential of ZYQL.

## 2 Materials and Methods

### 2.1 Materials and Reagents

The BCA protein assay kit (ZJ102), universal qPCR premix (MH101), and one‐step first‐strand cDNA synthesis premix (with dsDNase, MH102) were acquired from Epizyme Biotech (Shanghai, China). Glutathione S‐transferase activity assay kit (AKPR013M), superoxide dismutase activity assay kit (AKAO001M), reduced glutathione content assay kit (AKPR008M), lipase activity assay kit (AKFA006M), free fatty acid content assay kit (AKFA008M), and triglyceride content assay kit (AKFA003M) were obtained from Beijing Boxbio Science & Technology Co. Ltd. All other chemicals were of analytical grade, unless otherwise stated.

### 2.2 Preparation of TCM Ingredient Extracts (TCMIE)

Twenty grams of each of the 12 Chinese medicinal herbs used in ZYQL were weighed: *Angelica sinensis* (Oliv.) Diels (ASD), *Gardenia jasminoides* Ellis (GJE), *Kaempferia galanga* L. (KGL), *Lophatherum gracile* Brongn (LGB), *Chrysanthemum morifolium* Ramat. (CMR), *Lysimachia capillipes* Hemsl. (LCH), *Amomum villosum* Lour. (AVL), *Citrus reticulata* Blanco (CRB), *Santalum album* L. (SAL), *Eugenia caryophyllata* Thunb. (ECT), *Aucklandia lappa* Decne. (ALD), and *Lysimachia foenum‐graecum* Hance (LFH). These herbs were extracted with 200 mL of 60% ethanol at room temperature in the dark for 3 days. If the volume of the extraction solution was less than 200 mL, it was supplemented with 60% ethanol. The solution was then filtered, sealed, and stored in a dark place.

### 2.3 Lifespan, Pharyngeal Pumping Rate, and Motility Assays

*C. elegans* strains N2 were maintained according to the standard protocols, and lifespan assays were performed in the presence of FUdR (Cui et al. 2023). The synchronized N2 nematodes were transferred to NGM plates seeded with *E. coli* OP50 layers containing ZYQL or other TCMIEs. ZYQL and other TCMIEs were diluted to different 10% ethanol concentrations and then added to the plates. The plates were placed in the clean bench for 10 min to remove excess ethanol. The animals were transferred to fresh plates every 2 days. Survival curves represented the percentage of nematodes considered alive at given time points.

For pharyngeal pumping rate and motility assays, nematodes were transferred to a new plate after 4 days of treatment, and the number of pharyngeal contractions and body movements was counted under a stereomicroscope for 30 s.

### 2.4 Egg‐Laying Assays

The nematodes used for egg‐laying assays were prepared as follows: larval stage 4 nematodes (L4) were picked onto fresh plates seeded with *E. coli* OP50 and allowed to develop for 20 h at 20°C, and the resultant young adults were then used for assays. To test the response to drugs, individual young adults were transferred into wells of a 96‐well plate containing 100 μL of a solution of a particular TCMIE, and the number of eggs released at room temperature was scored after 90 min. Unless specified, the concentrations of drugs in M9 buffer were as follows: 5‐HT, 1 and 2 mg/mL; fluoxetine, 0.5 and 1.0 mg/mL; ZYQL, 0.5% and 1% Vol; TCMIEs, 1% Vol. Controls in M9 buffer (containing 1% Vol ethanol) alone were performed on each strain during each assay. Assays for all strains were replicated by three researchers in the lab, with each assay testing 30 animals.

### 2.5 RNA‐Sequencing Analysis

Nematodes (about 300) were prepared for transcriptomic analysis after being treated with fluoxetine for 2 days. Total RNA from the control and fluoxetine‐treated nematodes was extracted using the RNA extraction kit (RN07; Aidlab, Beijing, China). The quality and quantity analysis of total RNA, DGE library construction, sequencing, and data processing were performed on the Illumina Xten gene sequencing platform (Sangon Biotech, Shanghai, China). The DEGseq1.26.0 package was used for normalization and identification of differentially expressed genes (DEGs). Genes with an FDR < 0.05 and fold change ≥ 1.5 were considered DEGs. Bioinformatic analysis of RNA sequencing was conducted as described previously (J. Sun et al. 2023).

### 2.6 Quantitative Real‐Time PCR (qRT‐PCR) Analysis

Total RNA of nematodes was isolated by lysing samples in 250 μL of AIDzol Reagent (RN65, no chloroform) through freeze‐cracking. The total RNA was then subjected to reverse transcription into cDNA using a reverse transcription kit (Epizyme Biotech, Shanghai, China). qRT‐PCR was performed in triplicate with specific primers using SYBR Green Premix (Epizyme Biotech, Shanghai, China). Results were normalized to the *gapdh* gene according to the 2^−ΔΔCt^ method. Specific primers were included in Table S1.

### 2.7 Determination of Fat Metabolism and Lipase Activity in Nematodes

#### 2.7.1 Nile Red Staining of *C. elegans*

Synchronized L4 animals were bred on NGM plates with different drugs. Two days later, nematodes were collected and washed three times using M9 buffer with 0.01% Triton X‐100. Then, 1 mL of 40% isopropanol was added and incubated at room temperature for 3 min. In the dark, 1 mL of the Nile Red working solution (30 μg/mL in 40% isopropanol) was added to each sample and incubated for 2 h. The samples were then centrifuged at 1000 × *g* for 1 min to remove the supernatant. Subsequently, the worm suspension was placed on a microscope slide for imaging. Red fluorescence was visualized using a confocal microscope (FV1000; Olympus, Japan), and image analyses were performed using the ImageJ software.

#### 2.7.2 Triglyceride, Free Fatty Acid, and Lipase Activity

The synchronized L4 animals after the drug treatment were washed five times with M9 buffer immediately and then frozen for 10 min at −80°C, ground with a tissue grinder to prepare a homogenate, and finally centrifuged for 8 min at 10,000 × *g*. The protein content of the supernatant was determined using the BCA protein assay kit (Epizyme Biotech, Shanghai, China). The triglyceride (TG) and free fatty acid (FFA) content and lipase (LPS) activity in the nematodes were measured. The absorbance values of the samples were measured at specific wavelengths of 420, 550, and 710 nm, respectively.

### 2.8 Determination of Antioxidant System in Nematodes

The activities of superoxide dismutase (SOD) and glutathione S‐transferase (GST), as well as the content of reduced glutathione (GSH), were determined using commercial kits. After preparation and reaction, the absorbance of the samples was detected at specific wavelengths.

### 2.9 Statistical Analysis

All experiments included three biological replicates for each treatment, with the results presented as means ± standard deviation. One‐way analysis of variance and Tukey's test were performed using the SPSS software (version 20.0; SPSS Inc., Chicago, IL, USA). Confidence levels for statistical significance were set at *p* < 0.05.

## 3 Results

### 3.1 ZYQL Influences the Egg‐Laying Behaviors of *C. elegans*

**FIGURE 1:**
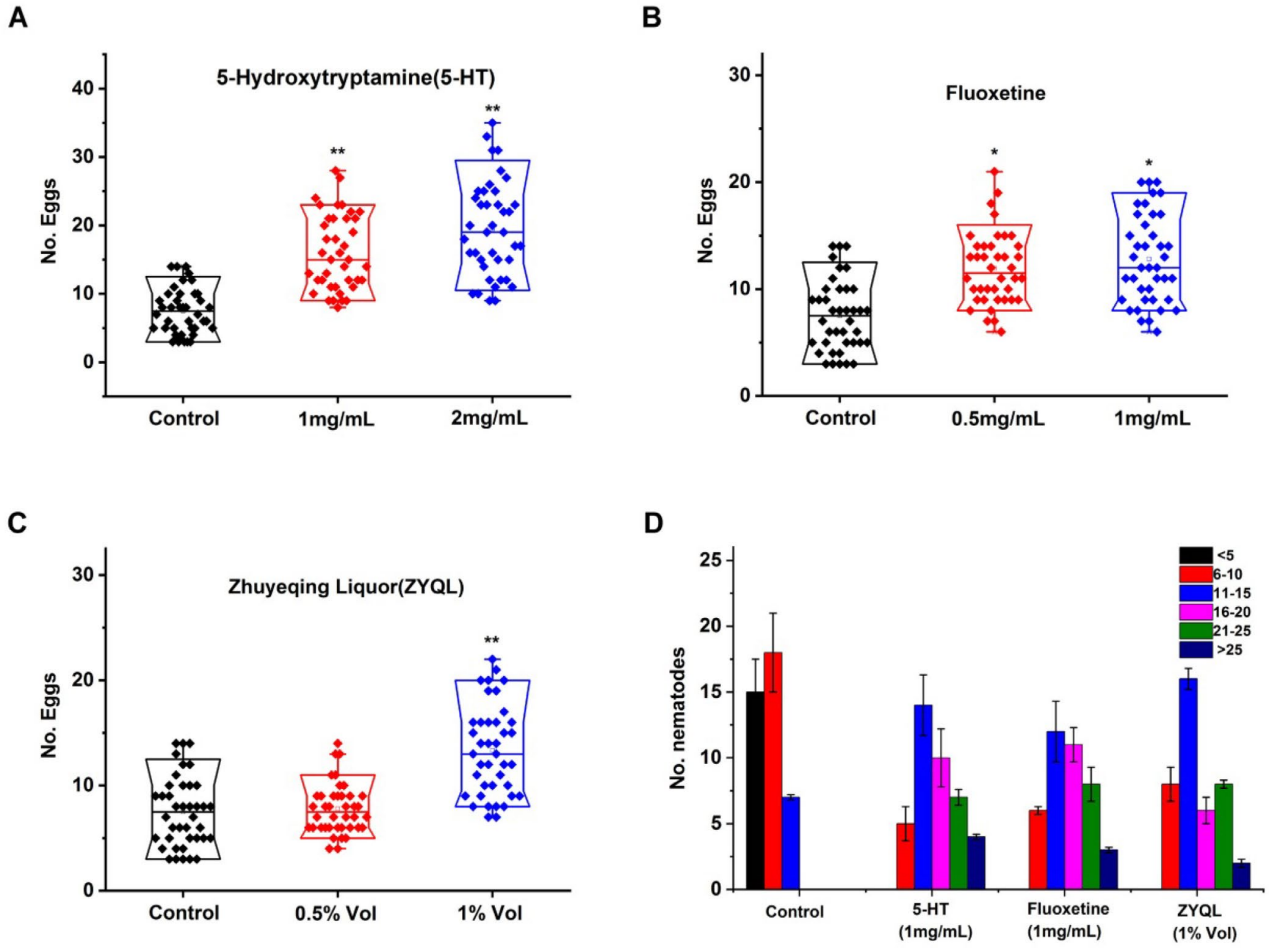
Egg‐laying induced by 5‐HT, fluoxetine, and ZYQL in *C. elegans*. Larval stage 4 animals were placed in 96‐well microtiter plates containing 100 μL of 5‐HT (1 and 2 mg/mL), fluoxetine (0.5 and 1 mg/mL), or ZYQL (0.5 and 1% Vol), and the number of eggs laid was counted after 90 minutes. *n* = 40 for each group. Egg‐laying induced by (A) 5‐HT; (B) fluoxetine; and (C) ZYQL; (D) the extent to which 5‐HT, fluoxetine, and ZYQL stimulated egg‐laying. *p < 0.05 and **p < 0.01 vs. control group.

Changes in egg‐laying behaviors are an important phenotype observed after the action of SSRI antidepressant drugs on nematodes (Dempsey et al. 2005). The increase in egg production in nematodes is largely related to the enhancement of 5‐HT signaling, which stimulates the muscle nerves that control ovulation (Kopchock et al. 2021). To confirm the antidepressant properties of ZYQL, the number of eggs laid was measured to determine the optimal ZYQL concentration that maintains egg‐laying behaviors, with exogenous 5‐HT and fluoxetine as positive controls. Figure 1A shows the effects of exogenous 5‐HT on the egg‐laying behaviors of *C. elegans* , indicating that the number of eggs increases at concentrations of 1 and 2 mg/mL. Fluoxetine showed a similar effect (Figure 1B). When nematodes were exposed to 1% Vol ZYQL, a similar effect on egg‐laying behaviors was observed (Figure 1C). In the presence of 1% Vol ZYQL, nematodes were stimulated to lay eggs to nearly the same extent as with 1 mg/mL 5‐HT and 1 mg/mL fluoxetine, suggesting that ZYQL may have antidepressant effects. The results indicate that 1% Vol ZYQL or 1 mg/mL fluoxetine is suitable for egg‐laying and growth of *C. elegans* , which was used in subsequent tests. From the distribution of nematodes, it is evident that all three types of active substances changed the interval distribution of the number of eggs laid. As shown in Figure 1D, the number of nematodes laying more than 16 eggs significantly increased. N2 nematodes were stimulated to lay eggs by ZYQL to nearly the same extent as by fluoxetine and 5‐HT. These results suggest that ZYQL, like fluoxetine and exogenous 5‐HT, can stimulate egg‐laying in nematodes. Furthermore, this result suggests that ZYQL or certain components in it may have antidepressant effects.

### 3.2 Six TCMIEs Used to Prepare ZYQL Can Stimulate Egg‐Laying Behavior in Nematodes

**FIGURE 2:**
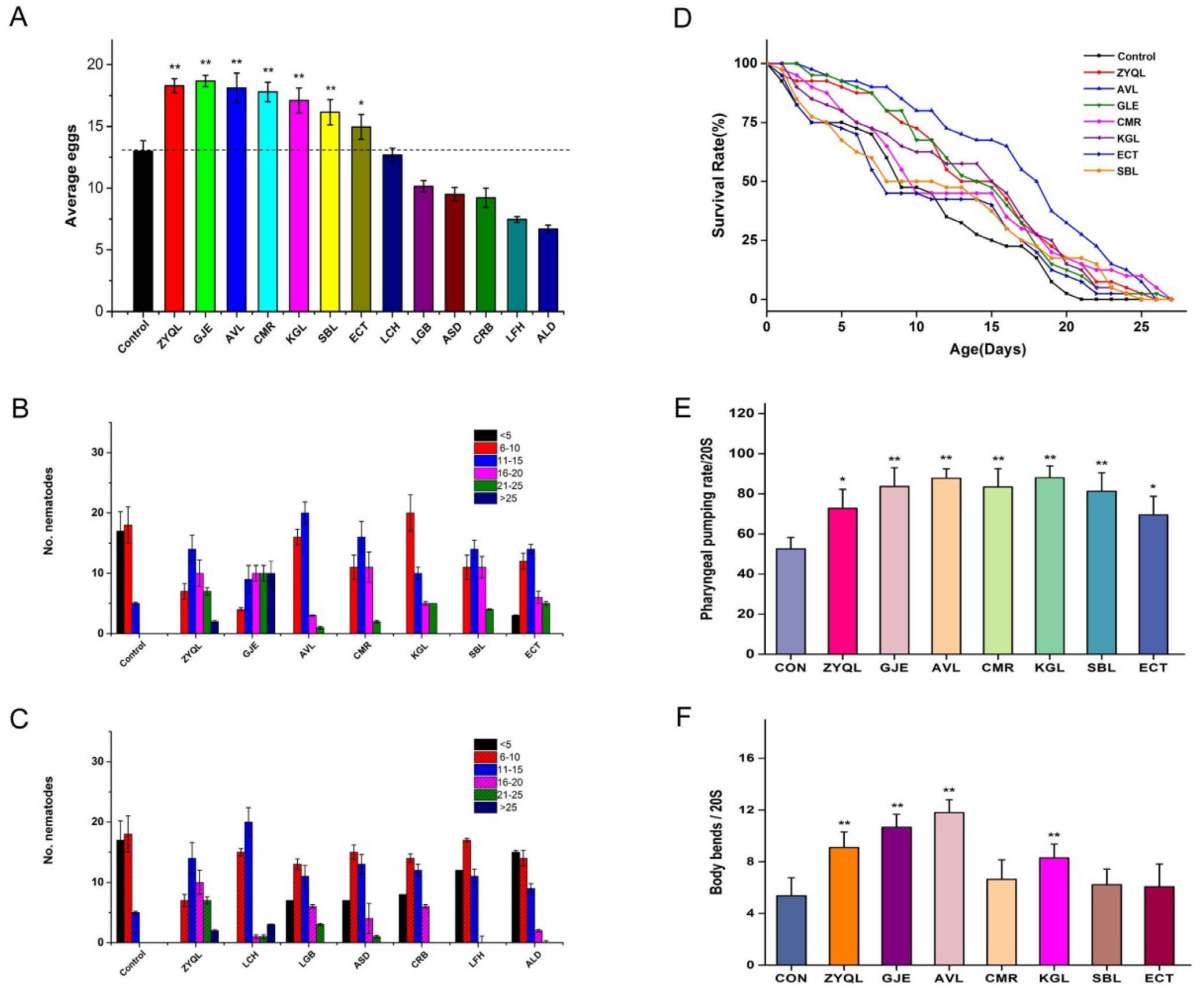
Egg‐laying, lifespan extensions, pharyngeal pumping, and body bends in *C. elegans* fed with ZYQL and TCMIEs. (A) Average number of eggs laid when nematodes are stimulated with ZYQL and TCMIEs; (B, C) extent of egg‐laying when the nematodes are stimulated with ZYQL and 12 TCMIEs; (D) lifespan extensions when nematodes are stimulated with ZYQL and 6 TCMIEs (that can stimulate egg‐laying in nematodes); (E) pharyngeal pumping; F, body bends. *p < 0.05 and **p < 0.01 vs. control group.

To determine the TCMIEs from medicinal herbs that affect the egg‐laying behavior of nematodes, L4 stage nematodes were treated with 12 TCMIEs under normal growth conditions. As shown in Figure 2A, the egg‐laying numbers of the ZYQL group and six specific TCMIEs were significantly higher than those of the control group. The extracts of GJE, AVL, CMR, KGL, SBL, and ECT stimulated the egg‐laying behavior of nematodes, while the extracts of LCH, LGB, ASD, CRB, LFH, and ALD inhibited or had no effect on the egg‐laying behavior.

Figure 2B,C show the extent of egg‐laying in nematodes. These results suggested that ZYQL can stimulate egg‐laying in N2 nematodes, and the extracts of GJE, AVL, CMR, KGL, SBL, and ECT stimulated egg‐laying to nearly the same extent as ZYQL. Conversely, the egg‐laying extent of the extracts from LCH, LGB, ASD, CRB, LFH, and ALD was similar to that of the control group. Figure 2D shows the survival curves of the extract‐treated groups, which shifted to the right compared to the control, indicating that these extracts prolonged the lifespan of the nematodes (*p* < 0.05). Among them, four TCM extracts, namely AVL, GJE, CMR, and KGL, showed statistically significant effects on prolonging the lifespan (*p* < 0.05) (Table S2). Although the extracts of ECT and SBL did not show statistical significance in prolonging the lifespan, they did not negatively affect the healthy lifespan of the nematodes.

The pharyngeal pumping rate and motility ability of nematodes were also analyzed. Compared to the control group, the number of pharyngeal contractions in nematodes treated with ZYQL was significantly higher, as was the case with nematodes treated with extracts of GJE, AVL, CMR, KGL, SBL, and ECT (Figure 2E). The number of body bends in nematodes treated with ZYQL was higher compared to the control group (*p* < 0.01; Figure 2F). Three TCMIEs, namely GJE, AVL, and KGL, had statistically significant effects on the body bends of nematodes (*p* < 0.05). After the action of ZYQL, there was no significant change in the body length and width of *C. elegans* (Figure S1). Taken together, ZYQL can stimulate egg‐laying and prevent the decline in motility and pharyngeal pumping rate.

### 3.3 Screening of Effective Antidepressant Biomarkers in *C. elegans*

#### 3.3.1 Mode of Action of Fluoxetine on *C. elegans* Through RNA‐Seq

**FIGURE 3:**
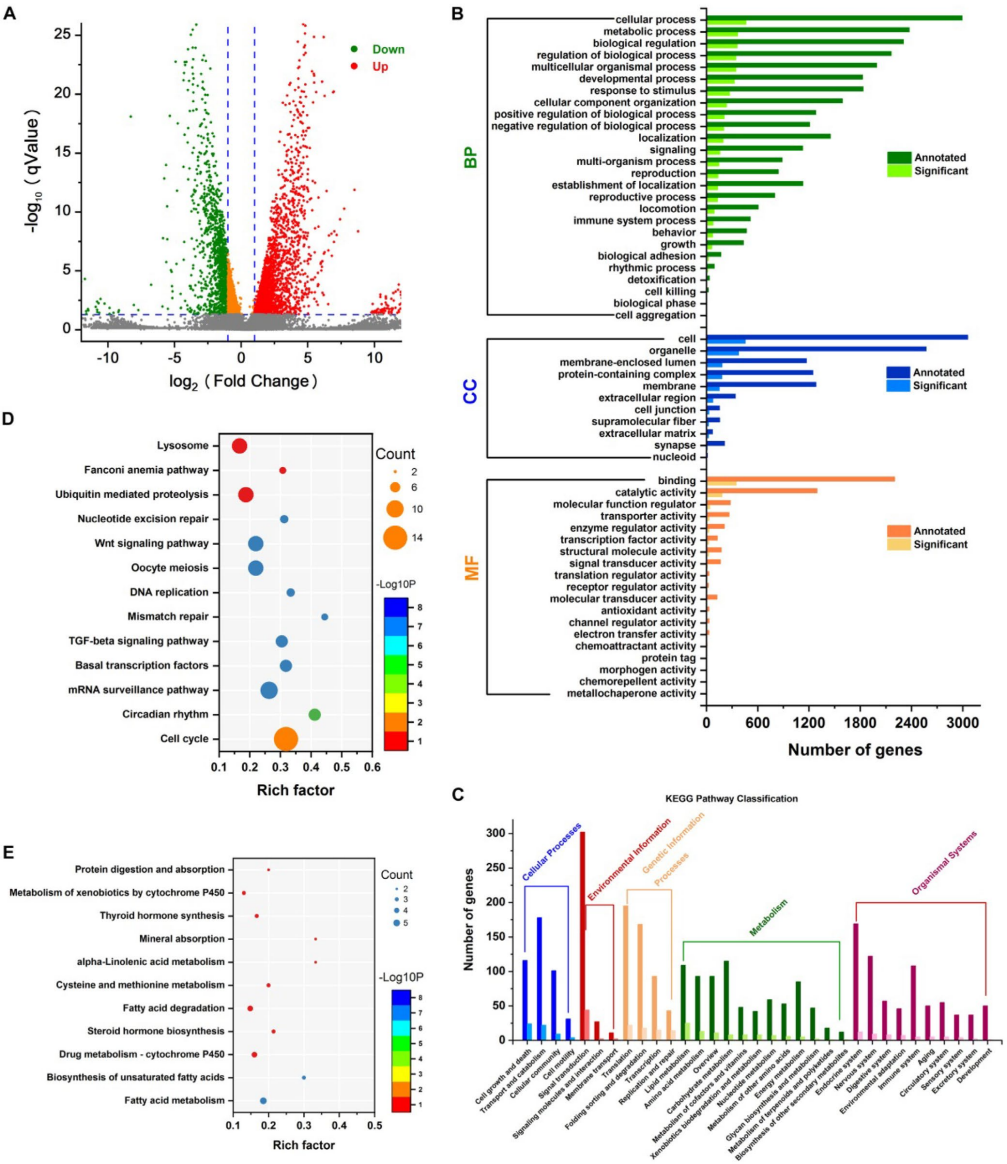
Transcriptome sequencing analysis of fluoxetine on the gene expression of *C. elegans* . (A) The volcano plot of DEGs in nematodes from the fluoxetine exposure groups; transcriptome annotation results from the (B) GO database and (C) KEGG database; KEGG enrichment analysis of (D) upregulated DEGs and (E) downregulated DEGs.

To identify suitable antidepressant biomarkers in nematodes, transcriptome analyses were used to explore the action mechanism of the antidepressant fluoxetine on the gene expression of *C. elegans* . A total of 40,134,770 (C1), 38,271,230 (C2), 53,192,594 (C3), 49,969,794 (F1), 36,417,602 (F2), and 39,255,360 (F3) clean reads were obtained after removing low‐quality sequences, adaptors, and empty reads. Based on the reference genome of *C. elegans* (GCF_000002985.6_WBcel235), 3600 DEGs were identified with a fold change (FC) > 1.5 and *Q* value < 0.05, including 2435 upregulated genes and 1165 downregulated genes (Figure 3A).

The potential functions of the DEGs were annotated using the Gene Ontology (GO) and KEGG databases. GO enrichment analyses of DEGs were performed, and these DEGs were divided into three categories: biological processes (BP), cellular components (CC), and molecular functions (MF). The results showed a total of 56 significantly enriched GO terms (including 26 BP, 11 CC, and 19 MF; Figure 3B). The BP category had the largest proportion. The three high‐ranking components of DEGs in this category were cellular processes, metabolic processes, and biological regulation. In the CC category, the highest proportions were found in the cell, organelle, and membrane‐enclosed lumen subcategories. Finally, in the MF category, the subcategory with the most DEGs was binding and catalytic activity.

#### 3.3.2 KEGG Pathway Analysis of DEGs

According to the KEGG classification, 329 significant genes were divided into 5 subcategories and 33 biochemical pathways (Figure 3C). These DEGs were involved in metabolic processes and organismal systems. Within the metabolic processes category, lipid metabolism, amino acid metabolism, and carbohydrate metabolism had the largest proportions. In the organismal systems category, the endocrine system and nervous system had the highest proportions. In addition, a large proportion of DEGs were found in pathways related to cell growth and death, transport and catabolism, and signal transduction (Figure 3C).

The up‐ and down‐regulated DEGs were further analyzed. As shown in Figure 3D,E, the up‐regulated DEGs in the fluoxetine groups were significantly enriched in pathways related to organismal systems. The cell cycle and oocyte meiosis pathways were activated; both are closely related to the egg‐laying behavior of nematodes (Figure 3D). In addition, the circadian rhythm pathway was activated, which is significant as disruption of the circadian rhythm is an important symptom in patients with depression. Fluoxetine promotes early secretion of melatonin in patients with depression, prolongs total sleep duration, corrects circadian rhythm disorders, and effectively alleviates depressive symptoms (Isa Kara et al. 2017). The TGF‐β and Wnt signaling pathways were also activated, which may be responsible for regulating egg‐laying and the cytoskeleton (Wong et al. 2013). Conversely, the down‐regulated DEGs were significantly enriched in metabolism‐related pathways (Figure 3E). This indicated that under fluoxetine exposure, the metabolic processes in the nematode were inhibited, including fatty acid metabolism, fatty acid degradation, biosynthesis of unsaturated fatty acids, *α*‐linolenic acid metabolism, and steroid hormone biosynthesis. These results also indicate that changes in fat or fatty acid metabolism may play a critical role in the treatment of depression with fluoxetine.

After delving deeper into the transcriptome data, it was found that the genes that underwent changes were mainly concentrated in the following pathways: cytoskeleton, fat metabolism, nervous system, cell cycle, antioxidant defense system, muscle contraction, immune inflammation, and carbohydrate metabolism. For the convenience of morphological observation and detection of biochemical indicators, the biomarkers from the cytoskeleton, fat metabolism, antioxidant defense system, egg‐laying, immune inflammation, and G protein‐coupled receptors were further validated.

### 3.4 Screening and Validation of Biomarkers

Combining the pathogenesis of depression with data research, we attempted to screen relevant biomarkers from the following aspects in nematodes. Meanwhile, two other common SSRI antidepressant drugs, sertraline and paroxetine, were used to validate the screened genes.

#### 3.4.1 Egg‐Laying

**FIGURE 4:**
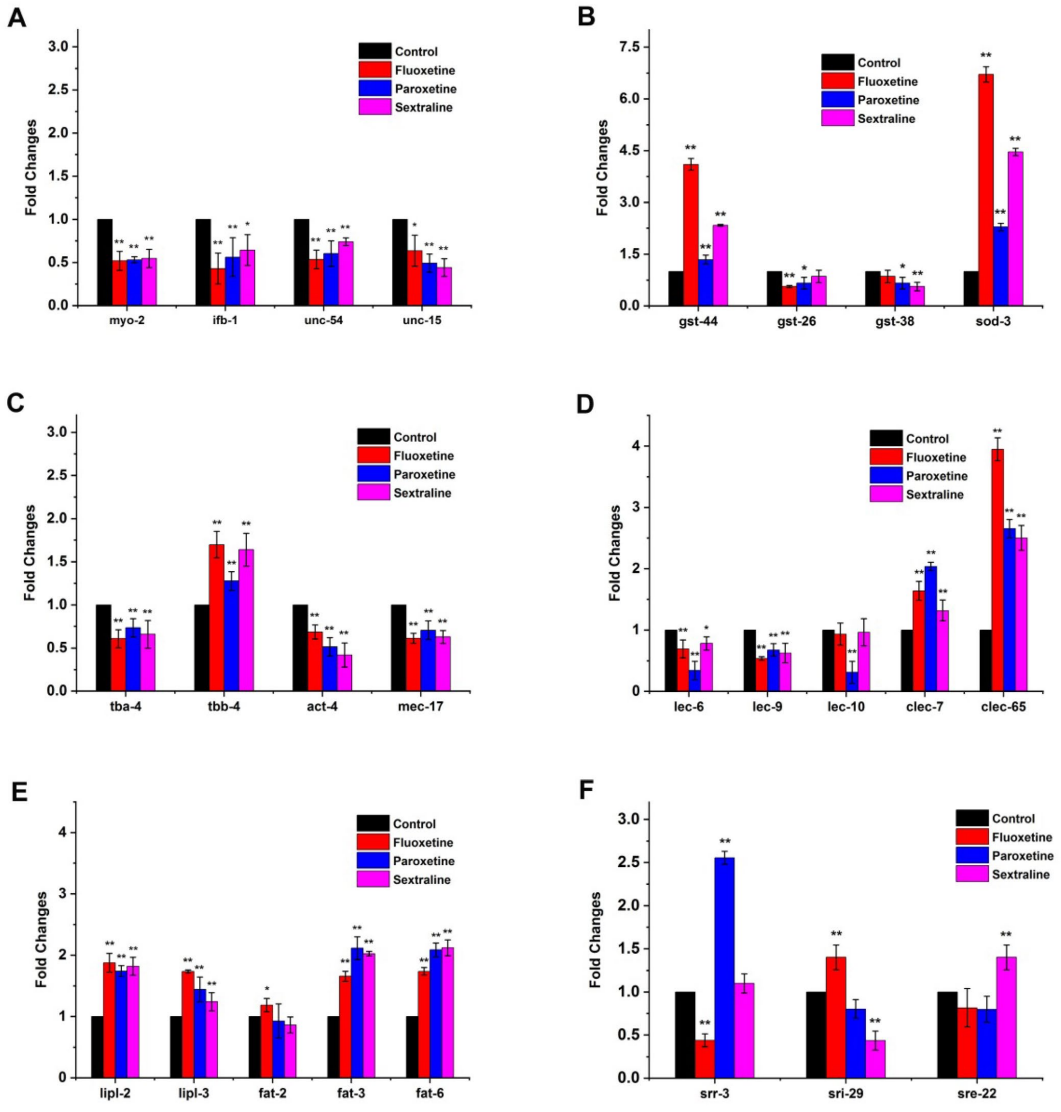
Screening and validation of biomarkers in *C. elegans* after treatment with SSRI antidepressants. (A) egg‐laying; (B) antioxidant defense system; (C) cytoskeleton; (D) inflammation‐related genes; (E) fat metabolism; (F) G protein‐coupled receptors. *p < 0.05 and **p < 0.01 vs. control group.

The egg‐laying behavior of nematodes is an important phenotype in the study of neural signal transduction, including depression (Dempsey et al. 2005). Four egg‐laying‐related genes from the significant DEDs, *myo‐2, ifb‐1, unc‐54*, and *unc‐15*, were selected as candidate biomarkers. These genes are involved in muscle contraction, device composition, and the regulation of oviposition behavior (Medrano and Collins 2023). As shown in Figure 4A, qRT‐PCR results indicate that the transcriptional level changes of these four genes are consistent with the transcriptome sequencing results for fluoxetine, all of which are down‐regulated. Under the action of paroxetine and sertraline, the transcription level changes to nearly the same extent as with fluoxetine (Figure 4A). Therefore, *myo‐2* and *ifb‐1* genes were selected for subsequent evaluation as biomarkers.

#### 3.4.2 Antioxidant Defense System

A large body of literature indicates a close relationship between oxidative stress and depression (Rybka et al. 2013). The intake of antioxidant supplements is associated with the improvement of depression and anxiety, confirming that antioxidant supplements can serve as potential adjunctive treatments for conventional antidepressants. Antioxidant‐related genes with the most significant differences were selected for evaluation, including *gst‐44, gst‐26, gst‐38*, and *sod‐3*. As evident from Figure 4B, the transcription levels of these genes are consistent after the administration of the three antidepressants, with a significant increase in transcription levels. Both *gst‐44* and *sod‐3* are associated with neurological diseases. The gene *gst‐44* is expressed in excretory cells and is involved in the response to reactive oxygen species. Human ortholog (s) of *gst‐44* are implicated in Alzheimer's disease and Parkinson's disease. The *sod‐3* gene is located in the mitochondrial respirasome and is involved in the removal of superoxide radicals, expressed in several structures, including the egg‐laying apparatus. Therefore, *gst‐44* and *sod‐3* were chosen as evaluation biomarkers.

#### 3.4.3 Cytoskeleton

Change of neuroplasticity is an important aspect of the pathological mechanism of depression, and cytoskeletal plasticity plays an essential role in supporting this neuroplasticity. The cytoskeleton (microtubules, microfilaments, intermediate fibers) endows cells with the ability to actively deform and resist passive deformation (Bianchi et al. 2003). The qRT‐PCR results in Figure 4C show that four genes (*tba‐4, tbb‐4, act‐4*, and *mec‐17*) have consistent responses to fluoxetine, sertraline, and paroxetine. Tba‐4 and act‐4 are structural constituents of the cytoskeleton, involved in microtubule and actin cytoskeleton organization. *Mec‐17* enables tubulin N‐acetyltransferase activity, and the loss of *mec‐17* leads to microtubule instability. Based on gene function, *tba‐4* and *act‐4*, two structural constituents of the cytoskeleton, were chosen as evaluation biomarkers.

#### 3.4.4 Inflammation‐Related Genes

The inflammatory process in the human body is a key response to resisting infection, and many researchers have found that inflammation is closely linked to the development of depression (Lopresti et al. 2014). Galectin‐3 (Gal‐3) mediates inflammation and host defense through cytokines, which are also associated with severe depression. Gal‐3 is a type of galactose‐binding lectin that serves as a pro‐inflammatory mediator and plays an important role in the body's inflammatory response, oxidative stress, and other processes (Pang et al. 2016). Inflammation‐related genes with the most significant differences were selected for evaluation, including *lec‐6, lec‐9, lec‐10, clec‐7*, and *clec‐65*. From Figure 4D, it can be seen that the transcription levels of these genes are consistent after the administration of the three antidepressants, with significant differences between *lec‐9* and *clec‐65*. Therefore, the genes of *lec‐9* and *clec‐65* were chosen as evaluation indicators.

#### 3.4.5 Fat Metabolism

There is a mutual influence and causal relationship between depression and obesity (Brennan et al. 2011). Five genes involved in fat degradation and fatty acid desaturation were selected as candidate screening biomarkers. The qRT‐PCR results showed that *lipl‐2, lipl‐3, fat‐3*, and *fat‐6* were consistent with the results of transcriptome sequencing, while the transcription levels of *fat‐2* varied under the action of the three drugs (Figure 4E). Both *lipl‐2* and *lipl‐3* encode lipase and are involved in the lipid catabolic process, with relatively low expression levels. Fat‐3 has stearoyl‐CoA 6‐desaturase activity and is involved in oviposition and pharyngeal pumping processes, while *fat‐6* has stearoyl‐CoA 9‐desaturase activity and is involved in innate immune response and long‐chain fatty acid biosynthetic process. Therefore, the genes *fat‐3* and *fat‐6* were chosen as evaluation biomarkers.

#### 3.4.6 G Protein‐Coupled Receptors

In mammals, many neurotransmitters that control behavior and emotions, such as 5‐HT, dopamine, γ‐aminobutyric acid, and glutamic acid, have corresponding G protein‐coupled receptors (Gururajan et al. 2016). In the transcriptome of nematodes treated with fluoxetine, many G protein‐coupled receptors change. As shown in Figure 4F, the levels of G protein‐coupled receptor proteins vary among the three SSRI antidepressant drugs, likely owing to differences in their molecular structures rather than the functional properties of SSRIs. *Srr‐3* is a gustatory receptor, *sri‐29* is a class I serpentine receptor, and *sre‐22* is a class epsilon‐2 serpentine receptor. Although the qRT‐PCR results were consistent with the transcriptome results for fluoxetine, the expression of these genes showed different trends after treatment with the three antidepressants. Therefore, these genes will not be considered in subsequent studies.

After transcriptomic analysis and qRT‐PCR validation, fluoxetine was found to act on nematodes, and 10 genes related to 5 types of factors influencing depression were screened. They were as follows: egg‐laying‐related genes: *myo‐2* and *ifb‐1*; antioxidant system‐related genes: *gst‐44* and *sod‐3*; cytoskeleton‐related genes: *tba‐4* and *act‐4*; fat metabolism‐related genes: *fat‐3* and *fat‐6*; and inflammation‐related genes: *lec‐9* and *clec‐65*.

### 3.5 Verification of the Components of ZYQL as Biomarkers of Depression

**FIGURE 5:**
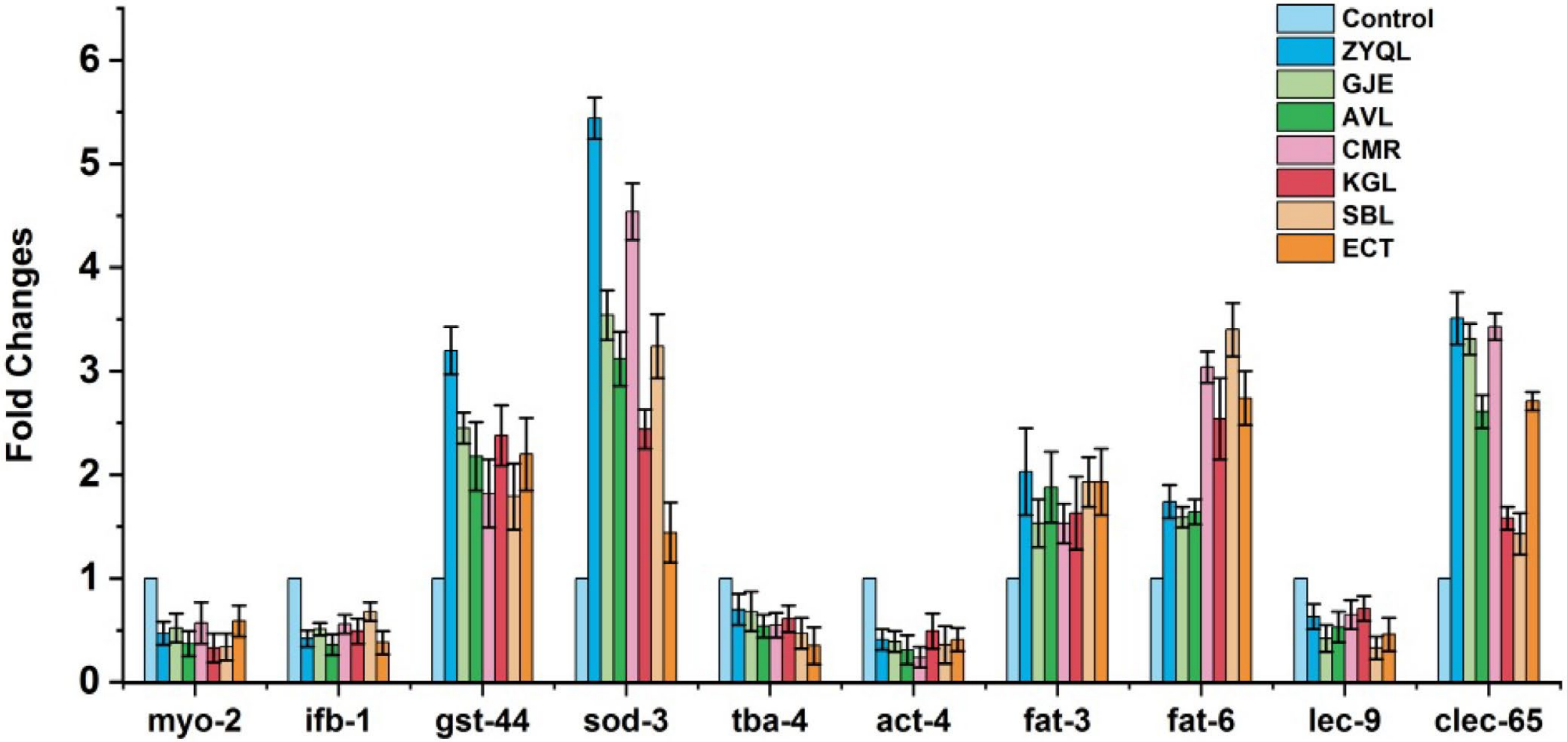
Evaluating the antidepressant activity of ZYQL and TCMIEs using selected biomarkers *p < 0.05 and **p < 0.01 vs. control group.

Synchronized L4 stage nematodes were transferred to NGM plates containing 10% Vol ZYQL, as well as other extracts. As observed in Figure 5, the results showed that *gst‐44, sod‐3, fat‐3, fat‐6*, and *clec‐65* were significantly increased in different groups, while the expression levels of *myo‐2, ifb‐1, tba‐4, act‐4*, and *lec‐9* were significantly reduced. These genes showed consistent responses to ZYQL, GJE, AVL, CMR, KGL, SBL, and ECT, and were also consistent with transcriptomic analysis results of fluoxetine, indicating that these genes can serve as biomarkers for screening SSRI antidepressants in *C. elegans* , and that ZYQL and its effective components, similar to fluoxetine, also have antidepressant functions.

### 3.6 Changes in the Fat Content of *C. elegans*

**FIGURE 6:**
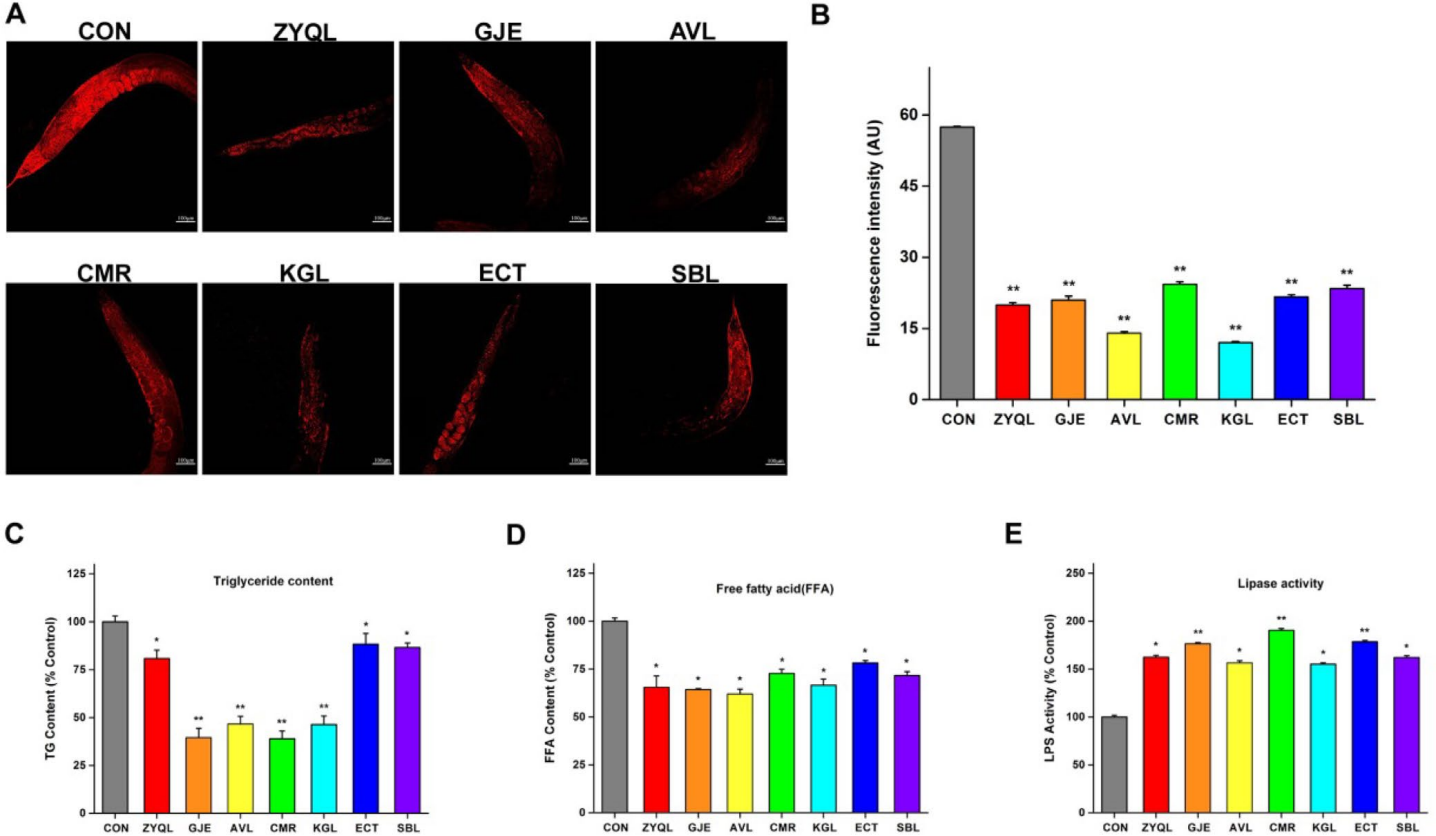
Changes in fat content in *C. elegans* after treatment with ZYQL and TCMIEs. (A) Nile Red staining to evaluate fat changes in nematodes; (B) quantitative analysis of fat content using Image J software; (C) triglycerides (TG) content; (D) free fatty acid (FFA) content; (E) lipase activity (LPS). *p < 0.05 and **p < 0.01 vs. control group.

Epidemiological data show that the relationship between obesity and depression is bidirectional (Almulla et al. 2023). Therefore, in antidepressant research on nematodes, the focus should be on changes in fat content and fat metabolism. The Nile Red staining method was used to observe changes in the body fat in nematodes following treatment with ZYQL and TCMIEs. Synchronized L4 stage nematodes were transferred to NGM plates containing 10% (v/v) ZYQL and TCMIEs. As shown in Figure 6A, it can be seen that compared with the control group, the fat content of nematodes was significantly reduced in the ZYQL group and other TCMIE groups. The fluorescence intensity of fat was reduced to 34.71%, 36.54%, 24.39%, 42.37%, 20.98%, 37.70%, and 40.79% compared to the control group (Figure 6B). Among these, AVL and KGL extracts had the greatest impact on reducing fat in nematodes.

After treatment for 2 days, TG, FFA, and LPS activity were determined. Compared to the control group, the TG content in nematodes fed with ZYQL and other TCMIEs decreased by 19.10%, 60.47%, 53.40%, 61.03%, 53.66%, 11.69%, and 13.46% (Figure 6C). The FFA content in nematodes fed with ZYQL decreased by 34.54%, 35.70%, 37.98%, 27.23%, 33.54%, 21.74%, and 28.35% (Figure 6D). The LPS activities increased by 62.46%, 76.47%, 56.50%, 90.53%, 55.18%, 78.46%, and 62.19% (Figure 6E).

### 3.7 Oxidative Stress and Antioxidation

High levels of free radicals are present in patients with severe clinical depression, leading to a long‐term state of oxidative stress. After antidepressant treatment, the levels of free radicals in these patients decrease, indicating a possible connection between the mechanism of depression and the oxidative antioxidant system (da Cruz Jung et al. 2019). In this experiment, nematodes were fed with ZYQL and TCMIEs, and the activities of SOD, GST, and GSH content were detected.

**FIGURE 7:**
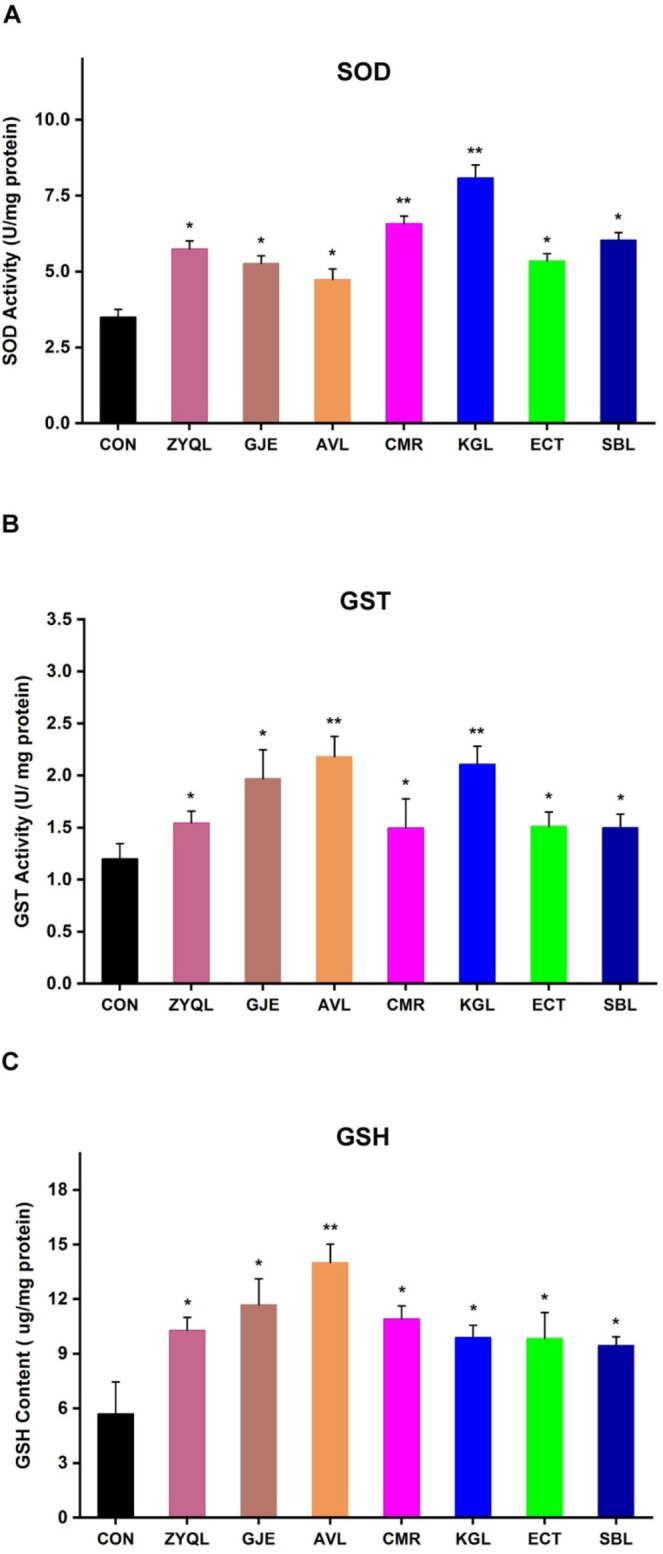
Changes in antioxidant defense system in *C. elegans* after treatment with ZYQL and TCMIEs. (A) SOD activity; (B) GST activity; (C) GSH content. *p < 0.05 and **p < 0.01 vs. control group.

SOD is a superoxide anion‐scavenging enzyme. As shown in Figure 7A, the results indicated that SOD activity in nematodes fed with ZYQL and other extracts was enhanced to varying degrees. The most significant increase in SOD activity was observed after treatment with the KGL extract. GST participates in primary metabolism, secondary metabolism, detoxification, and protection from oxidative damage. Compared to the control group, GST activities in nematodes fed with ZYQL and TCMIEs increased by 28.83%, 64.15%, 81.76%, 24.70%, 75.68%, 25.85%, and 24.85% (Figure 7B). The most significant increase was observed after treatment with extracts of KGL and AVL. The GSH content in nematodes fed with ZYQL and extracts increased by 80.46%, 105.04%, 145.95%, 91.46%, 73.43%, 72.54%, and 65.91%, respectively (Figure 7C). The most significant increase in GSH content was observed after treatment with extracts of AVL. After feeding nematodes with different drugs, the essential enzyme activities in the oxidative antioxidant system in nematodes increased, indicating that ZYQL and TCMIEs can enhance the in vivo antioxidant activity of nematodes.

## 4 Discussion

Biomarkers are indicators of normal biological processes, pathogenic processes, or specific therapeutic intervention (Group 2001). Depression is the most prevalent psychiatric disorder worldwide, imposing a significant economic and emotional strain on society (Trivedi 2020). To date, researchers have proposed biomarkers for depression from various perspectives, including imaging and electrophysiological studies, sleep and circadian rhythms, as well as metabolic, immune, genetic, and serum biomarkers (Gururajan et al. 2016; Kim et al. 2024). These biomarkers contribute to the diagnosis of depression and accelerate the novel drug discovery process. Owing to its many advantages, *C. elegans* has become a preferred model in many research fields, including neurodevelopmental studies (Ruszkiewicz et al. 2018). However, molecular markers of depression in nematodes have not been extensively studied.

SSRIs block 5‐HT reuptake and remove it from the synaptic cleft, increasing the concentration of 5‐HT in the synaptic cleft, allowing more 5‐HT to interact with its specific receptors for a longer duration and with greater intensity (Silva et al. 2012). This is the mechanism by which SSRIs exert their antidepressant effect. Fluoxetine, sertraline, and paroxetine are SSRIs that have been shown to induce behavioral effects at environmentally relevant concentrations in aquatic animals (Silva et al. 2012; Yamindago et al. 2021). The egg‐laying behavior of *C. elegans* has been used as a simple genetic system for identifying and characterizing the effects of antidepressant drugs on neurotransmitter pathways that generate and regulate specific behaviors (Bany et al. 2003; Weinshenker et al. 1995). Acute ethanol exposure produces rapid antidepressant‐like biochemical and behavioral responses in male C57BL/6NCrl mice (Wolfe et al. 2019). To eliminate the potential impact of ethanol, we used 1% Vol ethanol to mitigate this effect. By combining human depression biomarkers, 10 treatment‐response biomarkers in *C. elegans* were identified after fluoxetine treatment through transcriptomic analysis and qRT‐PCR validation (Figure 3). These markers reflect the physiological response of nematodes to the antidepressant drug fluoxetine from multiple aspects, including egg‐laying, the antioxidant system, the cytoskeleton, fat metabolism, and inflammation (Figure 3).

The egg‐laying behavior of nematodes is an important phenotype in the study of depression (Bany et al. 2003; Dempsey et al. 2005). 5‐HT modulates egg‐laying and locomotion behaviors of *C. elegans* (Riabinina et al. 2019). SERT antagonists, such as fluoxetine, paroxetine, and sertraline, could block 5‐HT uptake in vivo and increase 5‐HT concentration in the synaptic cleft. Therefore, 5‐HT can act on other targets, including hermaphrodite‐specific neurons (HSNs) (Medrano and Collins 2023). The *C. elegans* egg‐laying circuit comprises three parts: vulval muscles, HSNs, and VC motor neurons (Zhang et al. 2008). Among these, HSNs play a central role in driving egg‐laying by directly stimulating vulval muscles through the neurotransmitter 5‐HT (Zhang et al. 2008). The *C. elegans* gene mod‐5, which encodes a homolog of human SERT, is responsible for the reuptake of 5‐HT and its clearance from the synaptic cleft. The mRNA of mod‐5 did not show significant changes in the transcriptome (data not shown). In our study, the TGF‐β signaling pathway was upregulated (Figure 3D), and this pathway plays a key role in the sensory control of egg‐laying (Patterson and Padgett 2000). Meanwhile, the pathways of oocyte meiosis were also upregulated (Figure 3D), providing a basis for the improvement of nematode egg production. The gene *myo‐2* is a part of the muscle myosin complex and is expressed in neurons and the pharynx (Sadayappan et al. 2017). *Ifb‐1* is orthologous to human LMNA (lamin A/C) and LMNB2 (lamin B2) and is expressed in the egg‐laying apparatus. Therefore, these two genes can be preferred markers for evaluating the response to egg‐laying behavior in nematodes.

Oxidative stress and antioxidant defense biomarkers, including *gst‐44* and *sod‐3*, serve as another group of molecular markers in nematodes. Decreased SOD activity in red blood cells has been reported in patients diagnosed with recurrent depressive disorder (Rybka et al. 2013), and lowered serum SOD levels have been observed in patients with major depression (Stefanescu and Ciobica 2012). Gst‐44 enables glutathione dehydrogenase and glutathione transferase activities. The human ortholog (s) of *gst‐44* are implicated in Alzheimer's disease (Y. Chen et al. 2022) and Parkinson's disease (Allen et al. 2012). Glutathione S‐transferase omega‐1 (GSTO1) has been associated with the treatment response to SSRIs in the chronic mild stress depression model (Bisgaard et al. 2012).

Fat mass was positively associated with depression in the U.S. population (Zhu et al. 2024). Lipid metabolites related to depression may offer new prevention or treatment strategies for depression (C. Chen et al. 2018; Yang et al. 2023). Western diets contain higher levels of trans fatty acids and saturated fatty acids compared to long‐chain PUFAs and monounsaturated fatty acids (MUFAs), which may increase the risk of depression (Brennan et al. 2011; Jacka et al. 2010). Polyunsaturated fatty acids (PUFAs), including n‐3 and n‐6 fatty acids, are converted into very long‐chain fatty acids, such as eicosapentaenoic acid (EPA), docosahexaenoic acid (DHA), and arachidonic acid (Fernandes et al. 2017; Okereke et al. 2021). When transported to the brain, they influence the production of eicosanoids, which regulate inflammation. In *C. elegans* , *lipl‐2* and *lipl‐3* are involved in lipid catabolism. *Lipl‐3* localizes to the lysosome, while *lipl‐2* is expressed in the lumen of the gut and/or vesicles within the intestine (O'Rourke and Ruvkun 2013). In our study, *lipl‐2* and *lipl‐3* were upregulated 1.88‐ and 1.74‐fold after fluoxetine treatment (Figure 3E), respectively, indicating enhanced lipolysis activity.

*Fat‐3* and *fat‐6* are stearoyl‐CoA desaturases that catalyze the biosynthesis of MUFAs from saturated fatty acids. Fat‐3 is a delta‐6 desaturase that catalyzes the synthesis of long‐chain unsaturated fatty acids, including DHA (22:6n3) and EPA (20:5n3) (Hillyard and German 2009), while *fat‐6* is responsible for the conversion of C18:0 to C18:1Δ9 (Brock et al. 2007). After feeding nematodes with ZYQL and extracts, the FFA content decreased, and the enhancement of lipase activity also reduced the fat content in the nematode body (Figure 6E). In our study, increased biosynthesis of PUFAs and lipolysis jointly regulated fat metabolism. Fat metabolism and antioxidant biochemical indicators were evaluated after treatment with ZYQL. Saikosaponin improves cortical sphingolipid metabolism through apolipoprotein E, triggering neurovascular coupling and exerting antidepressant effects (Song et al. 2024). After treatment with ZYQL and other TCMIEs, the total fat content significantly decreased in nematodes (Figure 6A,B). The TG and FFA content also significantly decreased (Figure 6C,D). Meanwhile, the enhancement of lipase activity also reduced the fat content in the body of nematodes (Figure 6E). The synthesis and degradation of fat jointly regulate the reduction of fat content in nematodes.

Abnormal cytoskeleton leads to dendritic regression and reduced dendritic spine in patients with depression (Lee et al. 2002). Growing evidence has shown cytoskeleton‐related alterations in depression. Post‐translational modification (PTM) of actin and microtubules centrally influences cytoskeletal functions, and disturbance in these processes can cause cytoskeletal dysfunction associated with depression. These PTMs include actin arginylation (Saha et al. 2010), actin glutathionylation (Dalle‐Donne et al. 2005), microtubule acetylation (Bulinski 2007), and microtubule tyrosination (Bianchi et al. 2003). More importantly, downstream proteins in Wnt signaling pathways, such as MAP1B, directly affect microtubule stability and organization (Budnik and Salinas 2011). In our study, the Wnt signaling pathway was upregulated (Figure 3D). In our study, *tba‐4* and *act‐4* were downregulated after fluoxetine treatment (Figure 4C). Mec‐17, an alpha‐tubulin N‐acetyltransferase, was also downregulated. These results indicate that changes in the cytoskeleton can affect neuronal plasticity.

Major depressive disorder is associated with pro‐inflammatory markers, including C‐reactive protein (CRP), cytokines, neopterin, and tryptophan catabolites, which serve as diagnostic and treatment biomarkers in humans (Lopresti et al. 2014). However, these markers do not exist in nematodes. Gal‐3 is a novel biomarker with pro‐inflammatory properties, belonging to the family of β‐galactoside‐binding lectins (King et al. 2021). Gal‐3 is positively correlated with obesity and inflammation, as measured by inflammatory markers IL‐6 and CRP (Pang et al. 2016). Higher Gal‐3 levels are associated with higher levels of depressive symptoms, making Gal‐3 a potentially useful inflammatory biomarker for depression (Pang et al. 2016). Lec‐9 and clec‐65 enable carbohydrate‐binding activity and are suggested to contribute to immune specificity in both vertebrates and invertebrates (Schulenburg et al. 2008).

Using the 10 selected biomarkers, 6 TCMIEs from ZYQL were screened. These biomarkers showed responses nearly identical to those observed with fluoxetine treatment when *C. elegans* were treated with extracts of GJE, AVL, CMR, KGL, SBL, and ECT, indicating that these TCMIEs can serve as antidepressants (Figure 5). In addition, these six TCMIEs stimulated egg‐laying (Figure 2), reduced fat accumulation in nematodes (Figure 6), and enhanced the activity of the antioxidant system (Figure 7).

These six TCMs have been proven to have certain neuroprotective or antidepressant effects. Gardenia has been comprehensively studied for its antidepressant effects in animal models of MDD. Crocin is a natural carotenoid found in GJE that promotes adult hippocampal neurogenesis through the Wnt/β‐catenin signaling pathway (Tao et al. 2023), thereby exerting its antidepressant effect. Geniposide inhibits inflammatory response and modulates microglial polarization through the BTK/JAK2/STAT1 pathway, exhibiting antidepressant effects in LPS‐induced depressive mice (Zheng et al. 2021). Genipin exerts antidepressant‐like effects by regulating the 5‐HT and NE levels in the hippocampus in the forced swimming test and the tail suspension test in mice (Tian et al. 2010). Geniposide has a protective effect on chronic unpredictable mild stress‐induced depression in mice by activating the PI3K/Akt/GSK3β signaling pathway, reducing ceramide levels, and preventing hippocampal apoptosis (M. Wang et al. 2021). Kaempferol is a major flavonoid compound in edible plants (including *Kaempferia galangal* L.), which has antidepressant effects. It can enhance antioxidant abilities and anti‐inflammatory effects via upregulation of AKT/β‐catenin cascade activity in the prefrontal cortex of chronic social defeat stress mice models (W. Gao et al. 2019). ZYQL contains these TCM ingredients, enabling its antidepressant effect.

## 5 Conclusion

In summary, although biomarker measurement holds promise for psychiatric assessment, it is associated with numerous complexities. As demonstrated in this study, several inflammatory and oxidative stress biomarkers are associated with depression. However, none of these biomarkers possesses sufficient sensitivity and specificity to be used in isolation. This indicates the need for further research to identify alternative, more suitable single or collective biomarkers, as the existing results have been hampered by numerous inconsistencies and challenges across various studies.

## Author Contributions

**Xiaodong Cui:** funding acquisition (equal), methodology (equal), supervision (supporting), writing – original draft (lead). **Jiahui Shen:** data curation (equal), methodology (equal). **Jing Yan:** data curation (equal), formal analysis (equal), methodology (equal). **Chen Li:** supervision (equal), writing – review and editing (equal). **Jiao Li:** project administration (equal). **Fang Li:** supervision (equal), writing – review and editing (equal).

## Funding

The research was supported by applied basic research programs of Shanxi Province (No. 202203021211302) and the Open Project Program of Xinghuacun College of Shanxi (Shanxi Institute of Brewing Technology and Industry) (No. XCSXU‐KF‐202314).

## Conflicts of Interest

The authors declare no conflicts of interest.

## Supporting information


**Data S1:** fsn371349‐sup‐0001‐supinfo.docx.

## Data Availability

Data will be made available on request.
