# Supplementary material for: Evaluation of Antidepressant Effects of Zhuyeqing Liquor Using C. elegans as a Model
Source: Food Sci Nutr. 2025 Dec 15;13(12):e71349. doi: 10.1002/fsn3.71349 (PMC12704025; doi:10.1002/fsn3.71349)
Supplement: Supplementary file 1 — Data S1: fsn371349‐sup‐0001‐supinfo.docx. [file FSN3-13-e71349-s001.docx]

**Supporting information**

**Ta****ble S1 Sequences of qPCR Primers**

| Genes | Forward Sequence(5’ to 3’) | Reward Sequence(5’ to 3’) |
| --- | --- | --- |
| *myo-2* | ACATCGAGCAGGTCAACTGG | CGGCAATATCAAGCACACCG |
| *ifb-1* | CACAGGCACCAATGTCAGGAG | ACAAGGCCGGTGAAATTCCT |
| *unc-54* | GTACCGATGAGGCCGAGAAG | CAAGCCAGTTGAACACACGG |
| *unc-15* | ACAGAGTTGAACGTGAGCGT | TCTTGGTAGTCGAGGCAGGA |
| *lipl-2* | TCGGAAACTGCTTTCTGGCA | TCCTTTGCAAGGTGGGTGAA |
| *lipl-3* | TGTGCATCCACAGACTGGAC | TCCCAACTCCACTCCCAGAA |
| *fat-2* | GGTACCGGAGCTTCCATCAG | TGCGGATCCAACAATTCCCA |
| *fat-3* | GGCAAAATGGCAAACCGTGA | GTCCACTTGTGATGGCAACG |
| *fat-6* | GGCAAAATGGCAAACCGTGA | GTCCACTTGTGATGGCAACG |
| *tba-4* | GGTGGATCCTTCACCACGTT | CTCCTTGCCAACGGTGTAGT |
| *tbb-4* | TCTTCATGCCCGGATTTGCT | CAGCAACTGTCAGGTAGCGA |
| *act-4* | ACGAAGGATATGCCCTCCCA | TGACCTGTCCGTCAGGAAGT |
| *mec-17* | ACTCCACGTCGATTGGGAAC | ACAATGGCTTGCTCGACAGA |
| *gst-44* | CAACTTTGGAGCATGCGGAG | AAACTCTTCCAAACGCCGGA |
| *gst-38* | ACCGATGAGGAGTGGGAGAA | CCAAGCTTCACAGCGAGGTA |
| *gst-26* | ATGCTGGAAAAACCCCGGAA | GTGAGCCCATCTCCAACCAA |
| *sod-3* | AATCTACTGCTCGCACTGCT | TCCCTTTCGAAACAGCCTCG |
| *lec-6* | ATCTTCGCACTCCAGACGAC | ACCGTTGGACACAAACTCCA |
| *lec-9* | ATGGCCGCTCCAATTCTCAA | GTGGGAGATCTCGTTTCCCC |
| *lec-10* | TTCACCACTCGGAGCACTTC | ATCGACATGTCCGTCCCAAC |
| *clec-65* | AGCATCGCCAACTCTATCCG | TCGGGTACTTCTCGGGTCAT |
| *clec-7* | CGGTGTTGTCACATCTCCCA | CGGCACCAAGGTTCCACTAT |
| *srr-3* | GCGATGGTTTTTCGAGCAGG | TCTCACGCATTTCTGCGAGT |
| *sri-29* | TTCCTAGTGCTCACTGCTGC | GTTGGAAACAAGGCGACGAC |
| *sre-22* | AATCCCGTGTTCCTTGTCCC | CCTTGGTGGCAATTTGTGGG |
| *gapdh* | ACTGCTCGTCTTGAGAAGCC | CGAGACGAGCTTGACGAAGT |

**Table S2 Lifespan assay results and statistical analysis**

| Solvent | Maximum lifespan(d) | Mean lifespan(d±SEM) | n | *P* Value |
| --- | --- | --- | --- | --- |
| Control | 21 | 10.0±1.0 | 32 |  |
| 1% ZYQL | 26 | 14.3±1.1 | 35 | ＜0.01 |
| 1% AVL | 26 | 17.0±1.0 | 35 | ＜0.001 |
| 1% GJE | 27 | 13.0±1.2 | 30 | ＜0.05 |
| 1% CMR | 27 | 14.2±1.5 | 27 | ＜0.01 |
| 1% KGL | 26 | 13.7±1.2 | 31 | ＜0.05 |
| 1% SAL | 25 | 11.0±1.4 | 30 | n.s.(0.202) |
| 1% ECT | 25 | 12.6±1.5 | 26 | n.s.(0.055) |

**
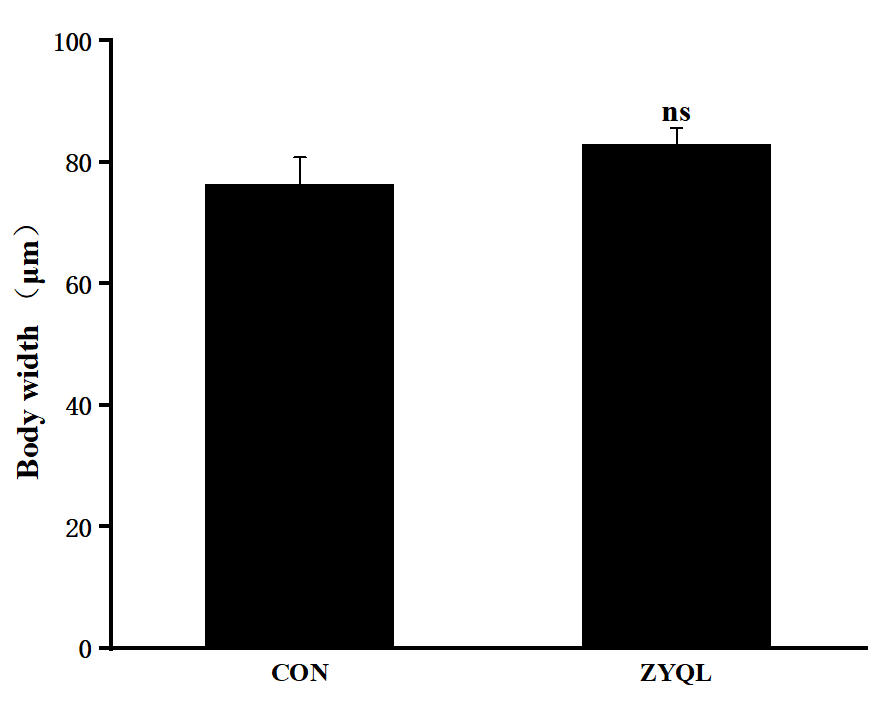

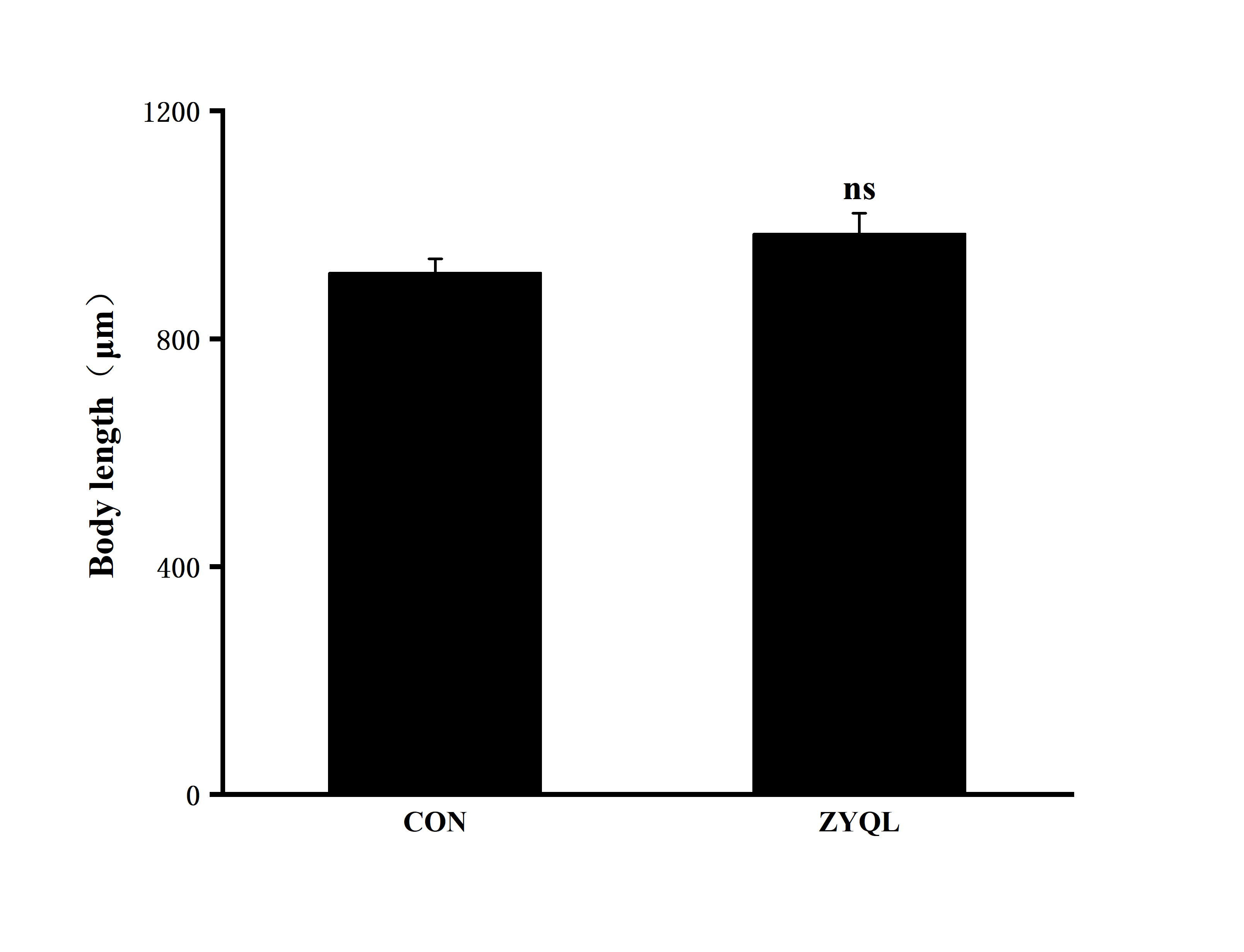
Figure S1**

**Fig. S1 The body length and body width of *C. elegans.***
